# Expression of miR-92a is associated with the prognosis in non-small cell lung cancer: An observation study

**DOI:** 10.1097/MD.0000000000030970

**Published:** 2022-10-14

**Authors:** Yu-Fang Huang, Ming-Wei Liu, Han-Biao Xia, Rong He

**Affiliations:** a Department of Respiratory and Critical Care, Suining Central Hospital, Suining, China; b Department of Emergency, First Affiliated Hospital of Kunming Medical University, Kunming, China.

**Keywords:** lymph node metastasis, miR-92a, non-small cell lung cancer, prognosis

## Abstract

With the development of molecular biology technology, the discovery of microRNAs (miRNAs) has provided new ideas for the diagnosis, treatment, and prognosis of lung cancer and laid a foundation for the study of this malignancy. To assess the potential prognostic value of miR-92a as a new biomarker in non-small cell lung cancer (NSCLC) via clinical evaluation, a total of 100 patients with NSCLC admitted to the Respiratory and Intensive Care Department of Suining Central Hospital in Sichuan Province between August 2007 and April 2020 were retrospectively analyzed. The correlation between miR-92a expression and prognosis of patients with NSCLC was also evaluated in the present study. The expression level of miR-92a was measured by PT-PCR and in situ hybridization. Chi-square test was adopted to explore the relationship of miR-92a expression and clinical features. Kaplan–Meier survival curves were plotted to delineate the overall survival rate of patients with NSCLC. Cox regression analysis was performed to evaluate the prognostic significance of miR-92a expression in NSCLC. The miR-92a expression in NSCLC tissue samples was significantly higher than that in normal lung tissues (*P* < .001) and significantly correlated with the Eastern Cooperative Oncology Group score, histological type, and distant metastasis (*P* < .05). Survival curve revealed that patients with NSCLC and high miR-92a expression had relatively higher mortality than those with low PAK4 expression (*P* = .001). Cox regression analysis explained that miR-92a expression was associated with the prognosis of patients with NSCLC (HR = 1.8, 95% CI: 1.0–3.2, *P* = .036). In summary, miR-92a was highly expressed in NSCLC tissues and could act as a prognostic factor for patients with NSCLC. These results illustrate that miR-92a expression plays an important role in the invasion and metastasis of NSCLC, and miR-92a can be used as a new biomarker to determine the prognosis of this cancer.

## 1. Introduction

The diagnosis and treatment of lung cancer have been updated and continuously improved. However, the mortality rate of this disease remains high because of the lack of specific clinical symptoms and signs during the early stages of lung cancer. Thus, patients are more likely to ignore it, resulting in a missed optimal treatment period.^[1]^ Some studies have shown that the 5-year survival rate of patients with early stage lung cancer after surgery is more than 70%, whereas that of patients with middle-advanced stage lung cancer is only approximately 20%. Therefore, the 5-year survival rate of patients with lung cancer is closely associated with early diagnosis and treatment.^[2,3]^

With the development of molecular biology technology, the discovery of microRNAs (miRNAs) has provided new ideas for the diagnosis, treatment, and prognosis of lung cancer and laid a foundation for the study of this malignancy. Clinical studies have found that compared with normal lung tissue, the expression levels of miR-21 in lung squamous cell carcinoma tissue, atypical hyperplasia tissue, and metastatic cancer tissue are increased by 9.1, 4.5, and 11.8 times, respectively,^[4]^ suggesting that miR-21 is closely related to lung squamous cell carcinoma. The occurrence and progression of cancer are closely related.^[4]^ Introducing exogenous miR-21 analogs into lung cancer H2170 cells to upregulate the expression of miR-21 in cells can lead to significantly higher cell proliferation ability in those with high miR-21 expression than those with low expression. miR-21 can promote the proliferation of cancer cells by inhibiting apoptosis and exert the function of oncogenes.^[5]^ miR-150 is highly expressed in non-small cell lung cancer (NSCLC) and is closely related to clinicopathological features, such as tumor node metastasis (TNM) stage, lymph node metastasis, and distant metastasis.^[6]^ Kaplan–Meier analysis showed that the 5-year overall survival rate of patients in the high expression group of miR-150 was 40.8%, whereas that in the low expression group was 69.2%, suggesting that high expression of miR-150 is associated with poor prognosis of patients.^[7]^ Recent studies have found that miR-92a promotes the proliferation, invasion, and migration of cervical cancer cells by directly targeting phosphoinositide 3 kinase regulatory subunit 1.^[8]^ Downregulated miR-18a and miR-92a inhibit the growth of NSCLC by targeting Sprouty 4.^[9]^ Current studies have found that miR-92a can be used as a marker for the diagnosis of colon cancer and is an important indicator for prognosis.^[10]^ However, whether miR-92a can be used as an important indicator for assessing the prognosis of NSCLC remains unknown. Therefore, the purpose of this study was to evaluate whether miR-92a can be used as a prognostic indicator for NSCLC.

## 2. Materials and Methods

### 2.1. Patients and tissue specimens

This study was approved by the Ethics Committee of Suining Central Hospital in Sichuan Province. All NSCLC tissue samples were collected on the premise that the patients signed informed consent forms.

**Figure 1.**
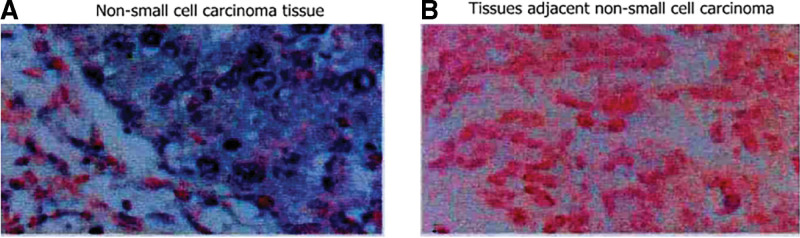
Comparison of the results from in situ hybridization staining between NSCLC tissues (A) and the tissues adjacent to the cancer tissues (B). The expression of miR-92a in the NSCLC tissues and the tissues adjacent to cancer. The positive signal of miR-92a is shown by the blue staining of the cytoplasm and red staining of the nucleus. NSCLC = non-small cell lung cancer.

Wax blocks of 100 surgically resected specimens were collected from patients with confirmed NSCLC in the Department of Respiratory and Critical Care Medicine, Suining Central Hospital between August 2007 and April 2018. The patient screening steps are shown in Figure 1. The patients included 78 men and 22 women aged 39–77 years (median, 61 years). Based on the 2002 version of the Union for International Cancer Control (UICC) standard, there were 38 stage I cases, 37 stage II cases, and 25 stage III cases. The samples included 57 cases of lymph node metastasis, 54 of squamous cell carcinoma, 46 of adenocarcinoma, 6 of highly differentiated cancer, 57 of moderately differentiated cancer, and 37 of poorly differentiated cancer. Thirty paracancerous normal lung tissue wax blocks were used as the control group. The inclusion criteria were as follows: patients who had not received any form of chemotherapy, radiotherapy, or targeted drug therapy; tissue specimens were obtained by puncture biopsy in all cases; paraffin-embedded sections of the specimens were excised during surgery; the pathological diagnosis was NSCLC; the patient had complete clinical data; and the acquisition and processing of specimens complied with the ethical norms and operating procedures of clinical trials. Patients with a clinical pathological staging of 0 or precancerous lesions were excluded from the study.

### 2.2. Experimental reagent

Quantitative real-time PCR (qPCR) kits were purchased from Thermo Fisher Scientific (USA). TRIzol reagents and RNA extraction kits were purchased from Beijing Solarbio Science and Technology Co., Ltd. (Beijing, China). miR-92a and U6 primers were synthesized by Shanghai Shenggong Biological Engineering Co., Ltd. (Shanghai, China).

### 2.3. Determination of miR-92a

Total RNA from the cell culture was extracted using the mirVana miRNA isolation kit, according to the manufacturer’s instructions. Revert Ace kits (TOYOBO, Osaka, Japan) were used to reverse transcribe the RNA samples into cDNA using different reverse transcribed primers. The primer sequences were as follows: U6, upstream 5-GCTTCGGCAGCACATATACTAAAAT-3, downstream 5-CGCTTCACGAATTTGCGTGTCAT-3; miR-92a, upstream 5-AGCTCTACGACTGTCACTCG-3, downstream 5-GTATGCATTCTATCGTAG-3′. The reaction conditions were as follows: 95°C for 10 minutes, 45 cycles of denaturation at 95°C for 10 seconds, and annealing and extension at 60°C for 60 seconds. The melting curve was determined. After the test was completed, the Ct value of each sample was automatically analyzed using a computer system, and 2−^ΔΔCt^ was used to calculate the relative expression of miRNA. The experiment was repeated three times.

### 2.4. In situ hybridization

The tissues were stored overnight at 65°C. The tissue was dewaxed by adding xylene twice for 10 minutes each. The tissue was rehydrated and treated with 30% acidic sodium sulfite at 50°C for 20 to 30 minutes. The nucleic acid hybridization rinse solution was used to rinse the tissue twice for 5 minutes each time. The samples were enzymatically digested and dehydrated. The sample was then immersed in acetone for 2 minutes, dried, placed on a glass slide, and covered at 56°C for 3 minutes, after which it was immersed in a denaturing solution at 73°C for 5 minutes. The following operations were performed under protection from light. The sample was subjected to gradient alcohol dehydration at −20°C, preheated at 45°C to 50°C for 2 to 5 minutes, and hybridized overnight with a probe at 42°C. The glass slide was washed with formamide and dried, and DAPI was added to the slide for double staining. The samples were observed under a fluorescence microscope after 20 to 30 minutes. The positive signal for miR-92a was determined by blue staining of the cytoplasm and red staining of the nucleus.

### 2.5. Clinical follow-up

The 100 enrolled patients were numbered based on their admission orders and hospital numbers to protect their privacy to the greatest extent. The patient’s personal phone number, email address, and other contact information were recorded by the HIS system based on the data registered when the patient was admitted to the hospital. When the patient was discharged from the hospital, the patient or the patient’s family was instructed to follow up at the outpatient clinic of our hospital at 1, 2, 3, and 6 months after discharge. After 1 to 3 years, the patient visited the hospital for follow-up every 6 months. After 3 years, the patient visited our hospital for follow-up every year. During follow-up, the patient’s current survival status was evaluated, including complications and secondary conditions if and when recurrence occurred. Detailed information was also recorded. The patient’s last follow-up was in January 2021. If the enrolled patient died or was lost to follow-up before October 2020, the overall survival time or disease-free survival (DFS) time was recorded as the time of death or loss to follow-up. The lost follow-up data were deleted during data analysis to control for confounding factors. In this study, none of the 100 patients with NSCLC was lost to follow-up.

### 2.6. Statistical analysis

SPSS19.0 was used to test the normality of the measurement data in the experiment. When the data conformed to a normal distribution, an independent-sample *t*-test was used. The Mann–Whitney test was performed with α = 0.05 as the test level. The results are expressed as median (interquartile range). To determine the diagnostic efficacy of lung cancer tissue miRNAs for NSCLC, the receiver operating characteristic (ROC) curve was used to analyze the samples. The Kaplan–Meier method was used to evaluate the survival of patients with lung cancer. Univariate and multivariate Cox proportional hazards models were used to analyze the importance of survival variables. The independent variables included in multivariate Cox analysis were statistically significant in univariate Cox analysis.

## 3. Results

### 3.1. Expression of miR-92a in the NSCLC tissues

The results of in situ hybridization staining showed that the expression of miR-92a in the patient’s NSCLC tissues was significantly higher than that in the adjacent tissues (Fig. 1).

### 3.2. Expression and clinical significance of miR-92a in the NSCLC tissues

The expression of miR-92a in NSCLC tissues was higher than that in adjacent lung tissues. The expression was also related to clinical parameters, such as vascular invasion, TNM staging, and lymph node metastasis (Table 1).

**Table 1:** Relationship between the expression of miR-92a in NSCLC tissues and clinical pathological parameters. The expression of miR-92a in the NSCLC tissues and tissues adjacent to the cancer tissues of the patients was measured by RT-PCR. The expression of miR-92a was expressed as mean ± standard deviation.

Clinicopathological parameters	n	Expression of miR-92a	*T* value	*P*
Age (yr)				
<60	34	1.78 ± 0.59		
≥60	66	1.82 ± 0.74	0.682	>.05
Gender				
Male	78	1.89 ± 0.71		
Female	22	1.81 ± 0.67	0.584	>.05
Organization type				
Squamous cell carcinoma	53	2.19 ± 0.84		
Adenocarcinoma	47	1.97 ± 0.51	0.869	>.05
Clinical staging				
Stage I	34	1.09 ± 0.32		
Stage II	38	1.39 ± 0.44	2.537	>.05
Stage III	28	2.69 ± 0.27	2.736	<.05
Lymph node metastasis				
No	42	0.79 ± 0.26		
Yes	58	1.91 ± 0.44	2.865	<.05
Differentiation				
Highly and moderately differentiated	62	1.07 ± 0.45		
Poorly differentiated	38	2.13 ± 0.92	3.278	<.05
Tumor size				
≥3 cm	54	2.24 ± 0.53		
<3 cm	46	1.98 ± 0.37	1.024	>.05
Organization source				
Lung cancer tissues	100	1.84 ± 0.76		
Normal lung tissue adjacent to cancer	30	0.45 ± 0.18	2.973	<.05

NSCLC = non-small cell lung cancer, RT-PCR = real-time PCR.

### 3.3. Effect of the expression of miR-92a on the prognosis of patients with NSCLC

**Figure 2.**
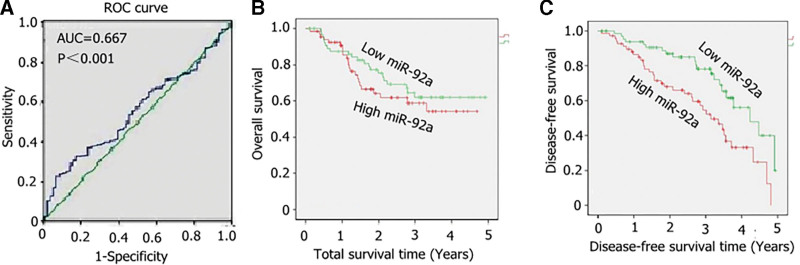
Changes in miR-92a in the lung tissues of patients with NSCLC and their effect on prognosis. The receiver operator characteristic (ROC) curve and Kaplan–Meier survival analysis were performed on the patients with NSCLC. (A) ROC curve. (B) Effect of miR-92a on the overall survival rate of patients with NSCLC. (C) Effect of miR-92a on the disease-free survival rate of patients with NSCLC. NSCLC = non-small cell lung cancer.

The area under the ROC curve (AUC = 0.669, *P* < .001) suggested that high miR-92a expression was correlated with NSCLC (Fig. 2A). Kaplan–Meier survival analysis was performed to explore the prognostic value of miR-92a in patients with NSCLC. The results showed that the overall survival (OS) of patients with high miR-92a expression was significantly higher than that of patients with low miR-92a expression (χ^2^ = 8.364, *P* = .003; Fig. 2B). However, there was no statistically significant difference in DFS between the two groups (χ^2^ = 0.831, *P* = .367; Fig. 2C). The abnormally high expression of miR-92a may have had an effect on the prognosis of patients with NSCLC (Fig. 2B and C).

### 3.4. Effect of the expression of miR-92a on the survival of patients with NSCLC

Univariate Cox analysis showed that lymph node metastasis (*P* < .0001), distant metastasis (*P* = .0435), tumor differentiation (*P* = .0472), TNM staging (*P* = .009), and miR-92a expression (*P* = .003) were significantly associated with the OS rate of patients with NSCLC (Table 2). Further study suggested an inherent connection between miR-92a and poor prognosis of patients with NSCLC.

**Table 2:** Correlation between miR-92a and clinical characteristics of patients with non-small cell lung cancer. According to the expression level of miR-92a in tumor tissues. We divided 100 patients with non-small cell lung cancer into miR-92a high expression group (n = 50) and miR-92a low expression group (n = 50). The clinicopathological parameters of 100 patients with non-small cell lung cancer were measured. The clinicopathological parameters was expressed as a percentage.

Clinicopathological parameters	N	Expression of miR-92a		χ^2^	*P*
		High expression (n = 50)	Low expression (n = 50)		
Age (yr)					
<60	34	16 (32%)	18 (36%)	0.000	1.000
≥60	66	34 (68%)	32 (64%)		
Gender					
Male	54	28 (56%)	26 (52%)	0.482	.628
Female	46	22 (44%)	24 (48%)		
Local invasion					
T1/T2	49	12 (24%)	37 (74%)	5.182	.039
T3/T4	51	38 (76%)	13 (26%)		
Organization type					
Squamous cell carcinoma	47	23 (46%)	24 (48%)	0.237	.769
Adenocarcinoma	53	27 (54%)	26 (52%)		
Tumor size					
≥3 cm	51	26 (52%)	25 (50%)	0.254	.712
<3 cm	49	24 (48%)	25 (50%)		
Lymph node metastasis					
No	51	16 (32%)	35 (70%)	4.892	.042
Yes	49	34 (68%)	15 (30%)		
Distant metastasis					
M0	39	21 (42%)	31 (62%)	2.2815	.084
M1	71	29 (58%)	19 (38%)		
Clinical staging					
Stage I/II	32	13 (26%)	32 (64%)	6.748	.028
Stage III/IV	68	37 (74%)	18 (36%)		

Statistically significant variables in univariate Cox analysis were included in multivariate Cox regression analysis, and the results showed that lymph node metastasis (HR = 4.5, 95%CI: 2.1–7.7, *P* < .0001) and miR-92a (HR = 1.8, 95% CI: 1.0–3.2, *P* = .036) were independent predictors of OS in patients with NSCLC (Table 3).

**Table 3:** Univariate and multivariate Cox analyses of the overall survival of patients with NSCLC. Cox analysis was performed on the effect of single factors, such as age, gender, and tissue type, on the survival rate of the patients with NSCLC. Statistically significant variables, namely, clinical staging, lymph node metastasis, degree of differentiation, and miR-92a expression, in the univariate Cox analysis were included in the multivariate Cox regression analysis.

Variable	Univariate Cox analysis HR (95% CI)	*P* value	Multivariate Cox analysis HR (95% CI)	*P* value
Age (yr)				
<60	1.3 (0.74–2.6)	.682	–	–
≥60				
Gender				
Male	1.4 (0.87–2.48)	.213	–	–
Female				
Smoking history				
Yes	0.97 (0.59–1.68)	.758	–	–
No				
Organization type				
Squamous cell carcinoma	1.1 (0.72–1.83)	.327	–	–
Adenocarcinoma				
Clinical staging				
I/II	2.2 (1.2–2.91)	.009	0.96 (0.53–2.7)	.869
III/IV				
Lymph node metastasis				
No	4.5 (2.4–8.5)	<.001	4.0 (2.1–7.7)	<.001
Yes				
Distant metastasis				
No	2.2 (1.0–4.6)	.0435	1.3 (0.61–2.5)	.792
Yes				
Differentiation				
Highly and moderately differentiated	2.5 (1.5–4.8)	.0472	1.6 (0.87–2.7)	.316
Poorly differentiated				
Tumor size				
≥3 cm	1.7 (0.87–2.39)	.219	–	–
<3 cm				
Expression of miR–92a				
High expression	2.4 (1.2–4.3)	.003	1.8 (1.0–3.2)	.036
Low expression				

NSCLC = non-small cell lung cancer.

## 4. Discussion

miRNAs are involved in biological processes such as cell division, proliferation, differentiation, apoptosis, development, metastasis, angiogenesis, and immune responses.^[11,12]^ In the process of tumor occurrence and development, miRNA not only plays an important role in promoting the tumor gene but also the tumor suppressor gene.^[13]^ Relevant studies have found a close relationship between the expression profile of miRNA and the embryonic origin of tumors, and the expression of miRNA is usually expressed in a tissue-specific manner.^[13]^ miRNAs are highly conserved, tissue-specific, time-sequential, and stable; their changes in organisms occur earlier than the appearance of protein markers.^[14]^

Studies have shown that the abnormal expression of miR-92a is closely related to the occurrence and development of tumors, and it plays an important role in the proliferation, apoptosis, invasion, and migration of various types of tumor cells.^[15,16]^ Recent studies have shown that the expression of miR-92a is upregulated in colon cancer, gastric cancer, and other tumors, which is related to tumor progression and angiogenesis.^[16–18]^ Zhou et al^[19]^ reported that the expression of miR-92a is upregulated in cervical cancer, and it can promote cancer cell proliferation and invasion by targeting F-box with 7 tandem WD40. Zhang et al^[20]^ reported that miR-92a induces colorectal cancer epithelial cell–mesenchymal transition to regulate cell proliferation, migration, and invasion by inhibiting phosphatase and tensin homolog expression. Ren et al^[21]^ showed that the upregulation of the expression of miR-92a in NSCLC can promote cancer cell proliferation, migration, and invasion; reduce cell apoptosis; and enhance chemotherapy resistance. The expression of miR-92a is inhibited, which showed the opposite effect. They confirmed that phosphatase and tensin homolog is a direct target of miR-92a. Studies have shown that the upregulated expression of miR-92a in colorectal cancer can promote tumor angiogenesis.^[17]^

Studies have shown that the abnormal expression of miRNAs is related to the occurrence and development of tumors, and its expression profile has obvious prognostic significance. In addition, miR-92a can inhibit the occurrence and development of hepatocellular carcinoma, gastric cancer, colorectal cancer, and other cancers.^[23]^ The characteristics of miRNAs as promoting genes for lung cancer tumors have been primarily studied in miR-17-92 clusters. These clusters also play an important role in the promotion of other tumors.^[11,25,26]^ miR-92a, a member of the miR-17-92 family, affects the migration of lung cancer cells via the TAT3→miR-92a→RECK axis.^[27]^ Some studies have also shown that high expression of miR-92a in serum is closely related to lymph node metastasis.^[28]^ Studies on NSCLC have shown that high miR-92a expression is related to NSCLC chemotherapy sensitivity and poor prognosis.^[21]^ Among the known miRNAs, miR-92a is one of the most attractive, and different institutions have shown that miR-92a has potential prognostic significance in patients with cancer.^[28]^ However, the results of different studies have been inconsistent. In this work, the overall risk of miR-92a in tumor prognosis was evaluated through clinical studies, thereby laying the foundation for subsequent studies and prognostic assessments of NSCLC. In our clinical studies, a comparison of the relative expression level of miR-92a in lung cancer tissues and normal tissues adjacent to the cancer tissues showed that the relative expression level of miR-92a in the lung cancer tissues was 1.84 ± 0.76, whereas the relative expression level in the adjacent normal tissues was 0.45 ± 0.18. The relative expression level of miR-92a in the lung cancer tissues was significantly higher than that in the adjacent normal tissues, and the difference was statistically significant (*P* < .05). The relative expression of miR-92a in the lung cancer tissues of patients with NSCLC was not related to their clinicopathological type (*P* > .05). In this study, the expression of miR-92a in patients with positive lymph nodes, poor tumor differentiation, and late clinical staging significantly increased. This result indicated that the invasion and metastatic potential of lung cancer were closely related to the high expression of miR-92a. Analysis of the ROC curve showed that the AUC of miR-92a was 0.613, sensitivity was 50%, and specificity was 68% (*P* = .083). Kaplan–Meier analysis showed that the median survival time of patients with NSCLC in the high miR-92a expression group was 15 months, whereas that in the low miR-92a expression group was 24 months. The difference between the two groups was statistically significant (*P* < .05).

Studies have shown that miR-92a and lymph node metastasis are independent risk factors for OS in patients with NSCLC. In this study, univariate Cox analysis of patients with NSCLC showed that lymph node metastasis (*P* < .0001), distant metastasis (*P* = .0435), tumor differentiation (*P* = .0472), TNM staging (*P* = .009), and miR-92a expression (*P* = .003) were significantly associated with the OS rate of patients with NSCLC. Statistically significant variables in univariate Cox regression analysis were included in multivariate Cox regression analysis. The results showed that lymph node metastasis (HR = 4.5, 95% CI: 2.1–7.7, *P* < .0001) and miR-92a expression (HR = 1.8, 95% CI: 1.0–3.2, *P* = .036) were independent predictors of OS in patients with NSCLC.

In summary, high expression of miR-92a is closely related to the poor prognosis of patients with NSCLC. Therefore, miR-92a could be used as a biomarker to assess the prognosis of these patients. In future studies, large sample sizes and high-quality studies are needed for further confirmation.

## Author contributions

Y-FH, ML, H-BX, and RH contributed to data acquisition and analysis. Y-FH and M-WL contributed to data interpretation.

H-BX and RH made contributions to the conception and design of the study and drafted the manuscript.

H-BX and RH made contributions to the design of the study and drafted the manuscript.

Y-FH, M-WL, and H-BX prepared Figures 1–3. Y-FH, M-WL, H-BX, and RH confirmed the authenticity of all raw data.

All the authors have read and approved the final manuscript.

**Conceptualization:** Yu-Fang Huang, Ming-Wei Liu, Rong He.

**Data curation:** Ming-Wei Liu, Han-Biao Xia.

**Formal analysis:** Ming-Wei Liu.

**Funding acquisition:** Han-Biao Xia, Rong He.

**Investigation:** Ming-Wei Liu, Han-Biao Xia.

**Methodology:** Ming-Wei Liu.

**Project administration:** Yu-Fang Huang.

**Resources:** Han-Biao Xia.

**Software:** Yu-Fang Huang, Han-Biao Xia, Rong He.

**Validation:** Yu-Fang Huang, Han-Biao Xia, Rong He.

**Visualization:** Han-Biao Xia, Rong He.

**Writing – original draft:** Ming-Wei Liu.

**Writing – review & editing:** Rong He.
